# Uterine biology in pigs and sheep

**DOI:** 10.1186/2049-1891-3-23

**Published:** 2012-07-16

**Authors:** Fuller W Bazer, Gwonhwa Song, Jinyoung Kim, Kathrin A Dunlap, Michael Carey Satterfield, Gregory A Johnson, Robert C Burghardt, Guoyao Wu

**Affiliations:** 1 Department of Animal Science, 442D Kleberg Center, 2471 TAMU, Texas A&M University, College Station, TX, 77843-2471, USA; 2 Department of Veterinary Integrated Biosciences, Texas A&M University, College Station, TX, 77843-2471, USA; 3 WCU Biomodulation Major, Department of Agricultural Biotechnology, Seoul National University, 1 Gwanak-ro, Seoul, Gwanak-gu, 151-742, Korea

**Keywords:** Genes, Growth factors, Interferon stimulated, Pregnancy, Pregnancy recognition, Uterus

## Abstract

There is a dialogue between the developing conceptus (embryo-fetus and associated placental membranes) and maternal uterus which must be established during the peri-implantation period for pregnancy recognition signaling, implantation, regulation of gene expression by uterine epithelial and stromal cells, placentation and exchange of nutrients and gases. The uterus provide a microenvironment in which molecules secreted by uterine epithelia or transported into the uterine lumen represent histotroph required for growth and development of the conceptus and receptivity of the uterus to implantation. Pregnancy recognition signaling mechanisms sustain the functional lifespan of the corpora lutea (CL) which produce progesterone, the hormone of pregnancy essential for uterine functions that support implantation and placentation required for a successful outcome of pregnancy. It is within the peri-implantation period that most embryonic deaths occur due to deficiencies attributed to uterine functions or failure of the conceptus to develop appropriately, signal pregnancy recognition and/or undergo implantation and placentation. With proper placentation, the fetal fluids and fetal membranes each have unique functions to ensure hematotrophic and histotrophic nutrition in support of growth and development of the fetus. The endocrine status of the pregnant female and her nutritional status are critical for successful establishment and maintenance of pregnancy. This review addresses the complexity of key mechanisms that are characteristic of successful reproduction in sheep and pigs and gaps in knowledge that must be the subject of research in order to enhance fertility and reproductive health of livestock species.

## Introduction

### Development of the conceptus (embryo/fetus and extra-embryonic membranes) and implantation

**Figure 1:**
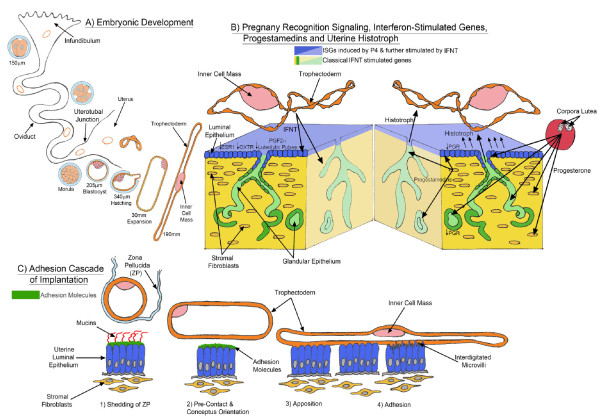
**[A] Oöcytes fertilized in the oviduct enter the uterus at the morula stage, hatch from the zona pellucida as spherical blastocysts and then transition to large spherical, tubular and filamentous conceptuses (embryo and its extra-embryonic membranes) with interferon tau (IFNT), the pregnancy recognition signal, being secreted from mononuclear trophectoderm cells between Days 10 and 21 of pregnancy.[B]** Endometrial epithelia cease expressing receptors for progesterone (PGR) due to autoregulation by progesterone and IFNT silences expression of receptors for estradiol (ESR1) and oxytocin receptors (OXTR) to abrogate development of the mechanism for oxytocin-mediated pulsatile release of prostaglandin F_2α_ (PGF) which would otherwise cause regression of the corpus luteum and cessation of progesterone secretion. The endometrial stromal fibroblasts express PGR and secrete fibroblast growth factor 10 that regulates uterine epithelia cell function. With down-regulation of PGR in uterine epithelia the uterine luminal (LE) and superficial glandular (sGE) epithelia express genes that are either induced by progesterone (P4) or induced by P4 and further stimulated by IFNT. Further, IFNT induces expression of interferon regulatory factor 2 (IRF2) in uterine LE and sGE to silence expression of classical interferon stimulated genes and allow expression of a unique set of genes that promote conceptus growth and development. The endometrial glandular epithelial cells (GE) and stromal fibroblasts do not express IRF2 and, therefore, express classical interferon stimulated proteins. Collectively, molecules secreted by uterine epithelia or transported into the uterine lumen by uterine epithelia form histotroph required for conceptus development. **[C]** The ovine conceptus undergoes the adhesion cascade for implantation.

As indicated in Figure 1, uterine receptivity and implantation of blastocysts for ruminants and pigs includes: 1) hatching from zona pellucida; 2) precontact and orientation of the blastocyst with uterine LE; 3) apposition between conceptus trophectoderm and uterine LE; 4) adhesion of conceptus trophectoderm to uterine LE and 5) no endometrial invasion by the conceptus [1].

#### Sheep

**Figure 2:**
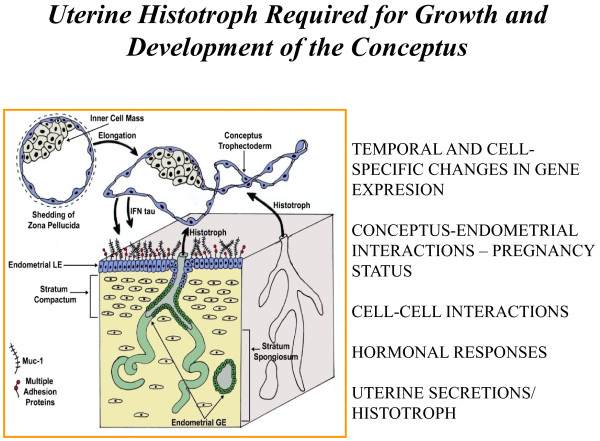
**Histotroph includes molecules secreted or transported into the uterine lumen to stimulate growth and development of the conceptus during the peri-implantation period and for the duration of pregnancy in species with epitheliochorial and syndesmochorial placentae.** The uterine luminal (LE) and superficial glandular (sGE) epithelia (blue color) in closest proximity to trophectoderm express novel genes in response to progesterone and interferon tau that support conceptus development in ewes. The uterine glandular (GE) and stromal cells express classical interferon stimulated genes during the peri-implantation period of pregnancy.

Sheep embryos enter the uterus on Day 3, develop to spherical blastocysts and then transform from spherical (Day 10, 0.4 mm) to tubular and filamentous conceptuses between Days 12 (1 mm × 33 mm), 14 (1 mm × 68 mm) and 15 (1 mm × 150–190 mm) of pregnancy with extra-embryonic membranes extending into the contralateral uterine horn between Days 16 and 20 of pregnancy [2]. Elongation of ovine conceptuses is a prerequisite for central implantation involving apposition and adhesion between trophectoderm and uterine luminal (LE) and superficial glandular (sGE) epithelia, hereafter designated as LE/sGE (Figure 1). There is then transient loss of uterine LE allowing intimate contact between trophectoderm and uterine basal lamina adjacent to uterine stromal cells to about Day 25 of pregnancy when uterine LE begins to be restored and placentation continues to Day 75 of gestation. All mammalian uteri contain uterine glands that produce/or selectively transport a complex array of proteins and other molecules into the uterine lumen and this is known collectively as histotroph (Figure 2). Uterine glands and the molecules that they secrete or transport into the uterine lumen are essential for conceptus development [3]. Components of histotroph required for elongation and development of conceptuses are transported into the uterine lumen via specific transmembrane transporters and receptors or they may be taken up by conceptus trophectoderm via pinocytosis [4-7]. Ewes that lacks uterine glands and histotroph fail to exhibit normal estrous cycles or maintain pregnancy beyond Day 14 [3].

Between Days 14 and 16, binucleate cells begin to differentiate in the trophectoderm and to migrate and fuse with uterine LE to form syncytia [8]. As indicated in Figure 1B, progesterone receptors (PGR) in uterine LE/sGE and GE are down-regulated after Day 13 of pregnancy which is associated with loss of expression of mucin 1, transmembrane (MUC1) and onset of expression of genes considered to be critical to conceptus development and implantation including glycosylated cell adhesion molecule 1 (*GlyCAM1*), galectin-15 (*LGALS15*), integrins and secreted phosphoprotein 1 (*SPP1*, also known as osteopontin) [7,9]. With apposition of the conceptus trophectoderm and uterine LE the filamentous ovine conceptus is immobilized in the uterine lumen and there is interdigitation of cytoplasmic projections of the trophectoderm cells and uterine epithelial microvilli to ensure maintenance of intimate contact [10]. Apposition of trophectoderm begins proximal to the embryonic disc and then spreads toward the ends of the elongated conceptus. The uterine glands are also involved in apposition as the trophoblast develops and extends finger-like villi or papillae into the mouths of the uterine glands to absorb components of histotroph between Days 15–20 after which time the papillae disappear [10-12]. The ovine uterine endometrium of ewes has both aglandular caruncular and glandular intercaruncular areas [13,14]. Synepitheliochorial placentation in sheep involves development and fusion of placental cotyledons with endometrial caruncles to form placentomes which are the primary sites of conceptus-maternal exchange for gases and micronutrients, such as amino acids and glucose.

#### Pig

After hatching from the zona pellucida, pig blastocysts undergo morphological transition to large spheres of 10 to 15 mm diameter and then tubular (15 mm by 50mm) and filamentous (l mm by 100 to 200 mm) forms between Days 10 and 12 of pregnancy and achieve a final length of 800 to 1000 mm between Days 12 and 15 of pregnancy [15]. During this peri-implantation period of rapid elongation, the trophectoderm produces significant amounts of estrogen (E2), as well as interferon gamma (IFNG) and interferon delta (IFND) [15-17].

Elongation of pig conceptuses during the peri-implantation period of pregnancy involves both a reduction in diameter and a rapid increase in length which is common to conceptuses of other livestock species in which conceptuses undergo elongation. Pig conceptus trophectoderm cells in the elongation zone are columnar, but they are cuboidal in areas peripheral to the elongation zone. This morphological difference is associated with changes in length and orientation of microfilaments in trophectoderm cells [18]. Orientation of microfilaments in trophectoderm cells change from horizontal to parallel relative to the lateral cell borders which suggests that elongation is initially through migration or concentration of trophectoderm cells into the same plane as the embryonic disc to form an elongation zone at around the 10 mm diameter stage of blastocyst development. Within the elongation zone, alterations in microfilaments and junctional complexes of trophectoderm cells and extension of filapodia from extra-embryonic endodermal cells allow movement and redistribution of cells toward the ends of tubular and then filamentous conceptuses. A summary of reported changes in the actin cytoskeleton of trophectoderm cells as they transition from spherical to tubular and filamentous forms is as follows: 1) cleavage stage embryos have filamentous actin concentrated at sites of contact between blastomeres; 2) compacting morulae accumulate actin at the margins of blastomeres; and 3) trophectoderm cells of expanding blastocysts initially exhibit pericellular distribution of actin that later forms continuous actin-rich lateral borders and stress fibers along their basal surface [19,20]. The actin cytoskeleton provides force generation for conceptus elongation as constricted regions along the length of filamentous conceptuses contain polarized trophectoderm cells with a distinct F-actin array. Focal adhesions are macromolecular complexes comprised of heterodimeric transmembrane integrin receptors that connect extra-cellular matrix (ECM) to the actin cytoskeleton to regulate cell growth, proliferation, survival, migration, gene expression, and cell morphology [21]. SPP1 binds integrin heterodimers of integrin subunits αv (ITGAV) and β6 (IGTB6) ITGAV:ITGB6 on trophectoderm and ITGAV and β3 (IGTB3):ITGB3 on uterine LE to induce assembly of focal adhesions that promote migration and attachment of trophectoderm cells to uterine LE critical to conceptus elongation and implantation [21].

### Concepts of tensegrity applied to conceptus elongation

Living cells use tensegrity (tensional integrity) architecture to control shape and structure of tissues and cells through changes in stability of cytoskeletal structures including: 1) microfilaments that are self-assembling actin polymers that form relatively rigid, but flexible networks that self-assemble into cross-linked bundles, or when associated with myosin II, form ‘contractile microfilaments’ that generate tension; 2) intermediate filaments that are polymers composed of cytokeratins in epithelial cells that form flexible cables extending from the cell surface to the nucleus to distribute force; and 3) microtubules that are larger hollow polymers of tubulin that extend across the cytoplasm to the cell periphery [22,23]. Integrins and associated proteins are ‘tensegrity structures’ because molecular connections between extra-cellular matrix (ECM), integrins, cytoskeletal filaments and nuclear scaffolds provide a discrete path for transfer of mechanical signals through cells as well as a mechanism for producing integrated changes in cell and nuclear structure. The resulting focal adhesion complex or ‘integrin adhesome’ [22] physically links integrins to the ends of contractile microfilament bundles (‘stress fibers’) to form a molecular bridge between ECM and cytoskeleton. Focal adhesions increase in size as tension increases across transmembrane integrin receptors [24]. Pulling on ECM tugs on integrins and associated focal adhesion proteins to deform the shape of molecules that elicit biochemical signals which change intracellular metabolism and gene expression. These mechanisms are likely responsible for elongation of conceptuses of pigs and sheep. They are also responsible for the substantial adaptive responses of the entire uterine wall to the increasing mechanical forces generated as a consequence of rapid fetal growth and accumulation of fetal fluids during gestation as discussed in a subsequent section.

In mice, leucine or arginine is required for formation of tight junctions for polarization of trophectoderm cells that allow them to pump water and other molecules into the blastocoel to expand blastocysts to large spherical forms and to allow migration and outgrowth of trophectoderm required for implantation [25,26]. Leucine and arginine can regulate migration and outgrowth of trophectoderm through activation of serine/threonine kinase mechanistic target of rapamycin (MTOR) cell signaling which activates Rac-1, a member of the Rho GTPase family. Increased MTOR cell signaling also stimulates protein synthesis and expression of insulin-like growth factor 2 (*IGF2*), nitric oxide synthases (*NOS*) and ornithine decarboxylase (*ODC1*) mRNAs [27,28]. Implantation of human blastocysts and migration of human extravillous trophectoderm requires multiple Rho GTPase family members in both trophectoderm cells and endometrial stromal cells into which they invade [29,30]. Rho GTPases including RhoA, Rac1 and cell division cycle 42 (CDC42) are ubiquitous proteins that control cytoskeletal changes by forming actin-containing stress fibers and projecting filopodia and lamellipodia during cell migration by linking ECM molecules with the actin cytoskeleton to assemble focal adhesions. Therefore, activation of GTPases may be controlled by integrin activation, but the mechanism(s) whereby ECM favors activation of individual molecules is not known [31].

The MTOR signaling pathway is linked to elongation of conceptus trophectoderm in sheep [32]. For ovine conceptus development during implantation and placentation, integrin activation by SPP1 binding and arginine are proposed to stimulate remodeling of trophectoderm for elongation and adherence to uterine LE/sGE via cytoskeletal reorganization that facilitates cell motility, stabilizes adhesion, and collectively activates MTOR signaling pathways mediated by protein kinase b-alpha (AKT1), tuberous sclerosis 1 and 2 (TSC1/TSC2) and MTORC1 (cell proliferation and mRNA translation), as well as mTORC2 (cell migration, cell survival and cytoskeletal organization) in trophectoderm cells. For ovine trophectoderm cells, SPP1 binds ITGAV:ITGB3 and perhaps ITGA5:ITGB1 to induce focal adhesion assembly, a prerequisite for adhesion and migration through activation of: 1) ribosomal protein S6 kinase (RPS6K) via crosstalk between MTOR and MAPK pathways; 2) MTOR, phosphatidyl inositol kinase 3 (PIK3), MAPK3/MAPK1 (ERK1/2) and MAPK14 (P38) signaling to stimulate trophectoderm cell migration; and 3) focal adhesion assembly and myosin II motor activity to induce migration of trophectoderm cells. These cell signaling pathways, acting in concert, mediate adhesion, migration and cytoskeletal remodeling of ovine trophectoderm cells essential for expansion and elongation of conceptuses and attachment to uterine LE for implantation [32].

The importance of E2 to implantation of pig conceptuses is underscored by the fact that premature exposure of the pregnant uterus to estrogen on Days 9 and 10 results in degeneration of all pig conceptuses by Day 15 [33]. The leading candidate molecules for attaching trophectoderm to LE in pigs are SPP1 and its integrin receptors to induce cytoskeletal reorganization, stabilize adhesion, and transduce signals through numerous signaling intermediates [34]. SPP1 induced by conceptus estrogens in uterine LE directly adjacent to implanting conceptuses binds ITGAV:ITGB6 on porcine trophectoderm cells and ITGAV:ITGB3 on uterine LE cells to promote attachment of the conceptus to the uterus during implantation in pigs [35].

### Down-regulation of expression of receptors for estrogen (ESR1) and progesterone (PGR) receptors is a prerequisite for implantation in sheep and pigs

#### Sheep

Mechanisms regulating responses of the ovine uterus to endocrine and paracrine signals during the estrous cycle and pregnancy require tissue- and cell-specific regulation of expression of both ESR1 and PGR [36]. In pregnant ewes, ESR1 expression is low or undetectable in uterine epithelia between Days 5 and 15 of pregnancy, but may increase slightly between Days 15 and 25 of gestation. Expression of PGR ceases in uterine LE/sGE and GE of pregnant ewes after Days 11 to 13 of gestation. However, uterine stromal cells express PGR throughout pregnancy. Clearly, temporal and spatial changes in expression of ESR1 and PGR are critical to changes in uterine biology and the establishment and maintenance of pregnancy in ewes. Indeed, proliferation and morphogenesis of uterine epithelia require the absence of effects of E2 and progesterone (P4) on uterine epithelia and this is accomplished by down-regulation of ESR1 and PGR in uterine epithelia, while maintaining expression of PGR in uterine stromal cells throughout pregnancy when circulating concentrations of P4 are high.

#### Pigs

Changes in expression of ESR1 and PGR in uterine epithelia and stromal cells of the pig have been reported [37,38]. ESR1 is expressed by uterine stromal and epithelial cells on Day 1, but only epithelial cells between Days 5 and 15 in both cyclic pregnant gilts. ESR1 abundance then increases in uterine epithelia of cyclic, but not pregnant pigs, between Days 15 and 18 after onset of estrus to affect secretion of luteolytic pulses of prostaglandin F_2α_ (PGF). Epithelial and stromal cells of the pig uterus express PGR between Days 0 and 5 of the estrous cycle and pregnancy, but PGR are expressed primarily by stromal cells between Days 5 and 10, and only by stromal cells between Days 10 and 18 for both cyclic and pregnant pigs. Information on temporal and spatial changes in uterine expression of PGR in the pig uterus beyond Day 18 of gestation is not available.

Uterine receptivity to implantation is established by actions of P4 and, in some species, P4 regulates or is permissive to the actions of locally produced cytokines and growth factors including interferons, chorionic gonadotrophin (CG), prolactin (PRL) and placental lactogen (CSH1), homeobox transcription factors and cyclooxygenase-derived prostaglandins through autocrine and paracrine pathways [5,6,39-46]. A fundamental paradox of early pregnancy is that cessation of expression of PGR and ESR1 by uterine epithelia is a prerequisite for uterine receptivity to implantation, expression of genes by uterine epithelia and selective transport of molecules into the uterine lumen that support conceptus development. Thus, effects of P4 are mediated via PGR expressed in uterine stromal and myometrial cells by stromal cell-derived growth factors known as “progestamedins” [47,48]. In ewes, down-regulation of PGR in uterine epithelia is a prerequisite for the expression of genes for uterine secretions and transport of molecules into the uterine lumen that support conceptus development. Gene expression is induced by P4 in uterine GE; however, co-administration of E2 and P4 to ovariectomized ewes up-regulates expression of PGR in uterine GE which inhibits expression of genes such as *SPP1*[36,49].

Down-regulation of PGR is associated with down-regulation of mucin 1, transmembrane (*MUC1*) on uterine LE/sGE which is a prerequisite for uterine receptivity to implantation [39], as well as up-regulation of expression of *LGASL15SPP1* and insulin-like growth factor binding protein 1 (*IGFBP1*) by uterine LE/sGE that stimulate migration and attachment of trophectoderm cells to uterine LE. Further, silencing *PGR* gene expression in uterine epithelia allows P4 to act via PGR-positive uterine stromal cells to increase expression of progestamedins, e.g., fibroblast growth factors-7 (FGF7) and −10 (FGF10) and hepatocyte growth factor (HGF) in sheep uteri [50] or FGF7, HGF and retinoic acid in primates [44,51]. These progestamedins exert paracrine effects on uterine epithelia and conceptus trophectoderm that express receptors for FGF7 and FGF10 (FGFR2IIIb*)* and HGF (MET; proto-oncogene *Met*). Many genes are P4-induced and interferon tau (IFNT) stimulated in ovine uterine LE/sGE that lack both PGR and signal transducer and activator of transcription 1 (STAT1) [6]. Both progestamedins and IFNT can act via mitogen activated protein kinases (MAPK) and phosphoinositide-3 kinase (PI3K) cell signaling to affect gene expression and uterine receptivity to implantation [52]. All Type I IFNs bind the same receptor, but activate cell-specific signaling pathways to differentially affect gene expression in uterine LE/sGE versus GE and stromal cells. Cell-specific gene expression in the ovine uterus is due to expression of interferon regulatory factor 2 (IRF2), a potent inhibitor of transcription, only in uterine LE/sGE by IFNT [4]. This ensures that uterine LE/sGE in direct contact with trophectoderm of the conceptus express novel genes such as nutrient transporters, proteases and protease inhibitors, morphogens and adhesion proteins that directly support conceptus development and implantation (Figure 2).

Pig conceptuses secrete E2 between Days 10 and 15 for pregnancy recognition and to increase expression of genes in uterine LE which stimulate proliferation, migration, and adhesion of trophectoderm, as well as implantation and conceptus development [4]. In pigs, E2 induces IRF2 in uterine LE/sGE to restrict expression of classical interferon stimulated genes to uterine GE and stromal cells and allow uterine LE to express novel genes in response to E2, IFNG and IFND [53].

E2-stimulated genes localized to uterine epithelia in pigs include aldo-keto reductase family 1, member B1(*AKR1B1*)*,* beta 2 microglobulin (*B2M*)*,* CD24 antigen (*CD24*)*,* lysophosphatidic acid receptor (*EDG7), FGF7, IRF2,* myxovirus resistance 1, mouse, homolog of (*MX1*)*,* neuromedin B (*NMB),* swine leukocyte antigens (*SLAs 1, 2,3, 6, 7, 8*)*,* solute carrier family 5 (sodium/glucose cotransporter), member 1 (*SLC5A1*)*, SPP1,* and stanniocalcin (*STC1*) [54]. IGF1 is expressed by uterine LE and GE of cyclic and pregnant pigs and IGF1 receptors are expressed by cells of the endometrium and conceptuses, suggesting paracrine and autocrine actions of IGF1 [55].

The established dogma is that FGF7 is a stromal cell derived paracrine mediator of hormone-regulated epithelial growth and differentiation; however, there is novel expression of FGF7 by uterine LE, particularly between Days 12 and 15 of the estrous cycle and pregnancy in pigs [56]. FGF7 binds to and activates FGFR2IIIb expressed by uterine epithelia and conceptus trophectoderm. E2 increases *FGF7* expression, but only when administered with P4 that down-regulates PGR in uterine LE and GE. FGF7 increases cell proliferation, phosphorylation of FGFR2IIIb, the MAPK cascade and expression of urokinase-type plasminogen activator, a marker for trophectoderm cell differentiation [56]. From about Day 20 of pregnancy, FGF7 is expressed by uterine GE in pigs in response to P4 and perhaps E2 and it is presumed to affect uterine epithelia and conceptus development throughout pregnancy (G.A. Johnson, R.C.Burghardt and F.W. Bazer, unpublished results). The increased secretion of estrogens between Days 15 and 30 of pregnancy also increases expression of endometrial receptors for prolactin (PRLR), which allow prolactin (PRL) to affect uterine secretory activity [57-60].

### Interferon Tau (IFNT) signaling for pregnancy recognition in ewes

During the estrous cycles of ewes, uterine LE/sGE release luteolytic pulses of PGF that induce structural and functional regression of the corpus luteum (CL) or luteolysis. Luteolysis in subprimate mammals is uterine dependent with uterine epithelia responding to sequential effects of P4, E2 and oxytocin (OXT), acting through their respective receptors. P4 stimulates accumulation of phospholipids in uterine LE/sGE and GE that then liberate arachidonic acid in response to E2-induced activation of phospholipase A. The arachidonic acid released from phospholipids is metabolized via prostaglandin synthase 2 (PTGS2) and prostaglandin F synthase for secretion of PGF. On Days 13 to 14 of the estrous cycle, P4 suppresses expression of PGR which allows rapid increases in ESR1 and OXT receptors (OXTR) for E2 and OXT to act on uterine LE/sGE. The pulsatile release of OXT from the posterior pituitary gland and CL induces pulsatile release of luteolytic PGF from uterine LE/sGE resulting in structural and functional demise of the CL [61].

IFNT, the pregnancy recognition signal in ruminants, silences transcription of *ESR1* and, therefore, the ability of E2 to induced expression of the *OXTR* gene in uterine LE/sGE. This effect of IFNT abrogates development of the endometrial luteolytic mechanism that requires OXT-induced release of luteolytic pulses of PGF [5]. However, basal production of PGF is maintained or increased in pregnant ewes due to continued expression of PTGS2 in both the uterus and conceptus [62]. Silencing *ESR1* expression by IFNT also prevents E2 from inducing *PGR* in endometrial epithelia. The absence of PGR in uterine epithelia is required for uterine LE/sGE and GE to express P4-induced, as well as P4-induced and IFNT-stimulated genes [5].

### Progesterone-induced and IFNT-stimulated genes in ovine uterine epithelia

In addition to signaling pregnancy recognition in ruminants, IFNT, in concert with P4, regulates expression of genes in the ovine uterus in a cell-specific manner. IFNT induces uterine GE and stromal cells to express classical interferon stimulated genes (ISGs) that include *STAT1, STAT2IRF1, IRF9*, interferon-stimulated gene 15 (*ISG15), myxovirus resistance 1 (MX1)*, 2’,5’-oligoadenylate synthase 1(*OAS*)*,* and radical s-adenosyl methionine domain-containing protein 2 (*RSAD2*). However, classical ISGs are not expressed by uterine LE/sGE because IFNT induces expression of IRF2, a potent transcriptional repressor [8]. Therefore, uterine LE/sGE express novel P4-induced and IFNT stimulated genes via PGR- and STAT1-independent cell signaling pathway(s) that are critical for implantation and establishment and maintenance of pregnancy. The alternative cell signaling pathways stimulated by IFNT in ovine uterine LE/sGE include MAPK and PIK3 [52,63]. This mechanism allows uterine LE/sGE in direct contact with conceptus trophectoderm to express novel genes critical to conceptus development.

Progesterone is permissive to the actions of IFNT. Therefore, the absence of PGR in uterine LE/sGE appears to remove inhibition of expression of genes for which expression is regulated by a progestamedin(s) and IFNT to support implantation and conceptus development [6]. In ewes, effects of P4 appear to be mediated primarily by FGF10 and, perhaps secondarily by HGF [50]. Novel P4-induced and IFNT stimulated genes include solute carrier family 7 (cationic amino acid transporter, y + system), member 2 (*SLC7A2)*, cystatin C (*CST3)*, cathepsin L (*CTSL*), solute carrier family 2 (facilitated glucose transporter), member 1 (*SLC2A1*), hypoxia-inducible factor 1, alpha subunit (*HIF2A*)*,* and galectin 15 (*LGALS15*) that encode for secretory proteins and transporters that deliver molecules into the uterine lumen that are critical to conceptus development [6,7].

### Stromal cell-derived progestamedins mediate effects of P4 on uterine epithelia

The paradigm of down-regulation of PGR in uterine epithelia prior to implantation is common to sheep [6], pigs [38], rhesus monkey [44], women [64], and mice [41]. Implantation is prevented if uterine LE/sGE and GE express PGR. Progestamedins include FGF7, FGF10 and HGF that are known to regulate function of LE/sGE and GE and to be synthesized and secreted by PGR-positive stromal cells [48]. Uterine stromal cells of primates [44] express FGF7 in response to P4, but its endocrine regulation in myometrium, tunica muscularis of arteries and placenta is not known. FGF7 and FGF10 act via FGFR2IIIb whereas the receptor for HGF is encoded by MET (HGFR). Both FGFR2IIIb and HGFR are unique to epithelial cells [16]. HGF is expressed by fibroblasts and smooth muscle cells of reproductive tissues of rodents, humans, sheep and horse, including uterus, placenta and ovaries [4]. Both FGF7 and HGF act on epithelial cells to stimulate proliferation, migration and differentiation. Although FGF7 acts as a progestamedin, endocrine regulation of HGF expression in the adult uterus is not clear. The primate uterus and mouse ovary express HGF in response to E2, but effects of P4 and androgens on HGF expression have not been reported. FGF10, a stromal derived growth factor with similar activities to FGF7, affects development of lung, brain, and limbs [4]. In the adult uterus and uteri of neonatal lambs, P4 increases expression of FGF10 and MET [3].

**Figure 3:**
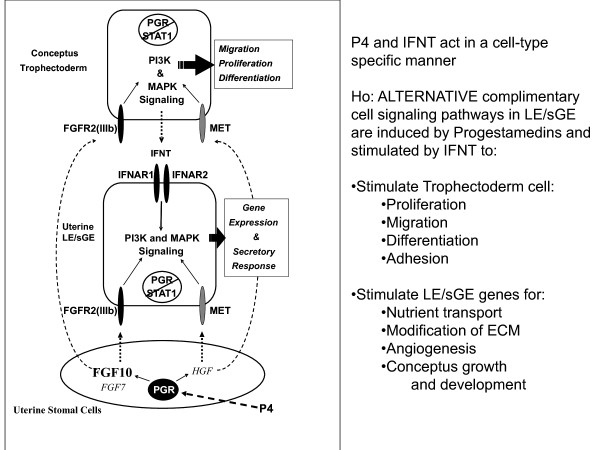
**Silencing expression of progesterone receptor (PGR) and signal transducer and activator of transcription facor 1 (STAT1) in uterine luminal (LE) and superficial glandular (sGE) epithelia is a prerquisite for implantation in mammals.** Therefore, progesterone acts via PGR-positive uterine stromal cells to increase expression of fibroblast growth factor-7 (FGF7) and FGF10, and hepatocyte growth factor (HGF) in sheep uteri. These progestamedins and interferon tau (IFNT) then exert paracrine effects on uterine epithelia and conceptus trophectoderm to stimulate gene expression and secretory responses by trophectoderm and uterine LE and sGE.

In adult ewes, *FGF10* mRNA is abundant in uterine stromal cells during the luteal phase of the estrous cycle and during the peri-implantation period of pregnancy when circulating concentrations of P4 are high (Figure 3). FGF10 is a candidate P4-induced progestamedin [3,50,65]. FGF10 is also expressed by chorioallantoic mesenchyme and FGFR2IIIb is expressed on adjacent trophectoderm suggesting that FGF10 mediates placental mesenchymal-trophectodermal interactions to stimulate development of the placenta [7]. FGF7 is expressed in media intima of uterine blood vessels of ewes which is consistent with its expression in spiral arteries of the primate endometrium [66,67]. However, FGF7 is not expressed by stromal cells proximal to LE/sGE and GE in ewes [66]. The nonoverlapping cell-specific patterns of expression for FGF10 and FGF7 in uteri of ewes suggest that these growth factors have independent roles in uterine functions and conceptus development.

HGF and HGFR are expressed in the ovine uterus during the estrous cycle and pregnancy (Figure 3). HGF is expressed by uterine stromal cells and *HGFR* mRNA is localized exclusively to LE/sGE and GE [67]. HGF is also expressed by chorioallantoic mesenchyme, and HGFR is expressed by trophectoderm. HGF may stimulate epithelial morphogenesis and differentiated functions required for establishment and maintenance of pregnancy, conceptus implantation and placentation [68]. HGF regulates human endometrial epithelial cell proliferation and motility [69] and mediates estrogen actions (i.e. estromedin) [70]. In pregnant ewes, HGF expression decreases between Days 11 and 13, increases from Day 13 to Days 15 and 17, and then decreases by Day 19. Expression of HGFR in pregnant ewes increases between Days 11 and 15, remains high through Day17, and then decreases by Day 19. The hormonal regulation of expression of HGF is unknown, but HGFR increases in the neonatal ovine uterine LE in response to P4 [3]. Expression of HGF in stromal cells of the ovine uterus is greatest when PGR are abundant in stromal cells, but absent in LE/sGE and GE. Similarly, HGFR expression increases in ovine endometrial epithelia when circulating levels of P4 increase and epithelial cell PGR decrease, implicating a role for P4 in regulation of abundance of HGFR, perhaps through P4-induced down regulation of PGR. Inflammatory cytokines such as interleukin one alpha (IL1A), IL6 and tumor necrosis factor alpha (TNFA) may also affect expression of HGF and HGFR [71]. Therefore, expression of HGF and HGFR may be coordinated by the actions of ovarian steroids and cytokines through a complex network. In mice, HGF is required for chorioallantoic mesenchymal-trophoblast interactions resulting in placental organogenesis [68]. In sheep, HGFR expression in trophectoderm and HGF expression in allantoic mesenchyme suggests similar roles for HGF in placental development and embryogenesis [67].

Early administration of exogenous P4 at 36 h after onset of estrus, i.e., about 6 h post-ovulation, advances conceptus development and IFNT secretion in both sheep and cattle. In this model P4-accelerates conceptus development and advances expression of uterine genes that favor survival and development of the conceptus [50,65,72,73]. In ewes, the early increase in circulating concentrations of P4: 1) advances the time of down-regulation of PGR in uterine epithelia and onset of secretion and abundance of IFNT in uterine flushings; 2) increases abundance of secreted proteins LGALS15, cathepsin L (CTSL), gastrin releasing protein (GRP), stanniocalcin (STC1), and IGFBP1 by uterine LE/sGE [50,65,74-78]; 3) increases expression of *FGF10* and, to a lesser extent, *HGFR* mRNAs [50]; 4) increases HGFR to increase responsiveness of uterine LE/sGE to HGF to enhance conceptus development since both FGFR2IIIb and HGFR are expressed by both uterine epithelia and trophectoderm [50,66,67]; and 5) decreases tight-junction associated proteins in uterine LE that may facilitate paracellular trafficking and/or transport of stromal and serum-derived molecules [72].

### Estrogen, prolactin and pregnancy recognition in pigs

Pig conceptuses begin secreting E2 on Days 11 and 12 of pregnancy which activates mechanisms to redirect PGF secretion away from the uterine vasculature (endocrine secretion) and into the uterine lumen (exocrine secretion). The endocrine:exocrine theory of estrogen-induced maternal recognition of pregnancy in pigs is based on evidence that: (i) the uterine endometrium of cyclic gilts secrete luteolytic PGF; (ii) pig conceptuses secrete estrogens which are antiluteolytic; (iii) PGF is secreted into the uterine vasculature (endocrine) in cyclic gilts for transport via blood to the ovary to induce CL regression (luteolysis); and (iv) secretion of PGF in pregnant gilts is into the uterine lumen (exocrine) where it is sequestered and metabolized to prevent it from being transported to CL to cause luteolysis [79]. PRL is also involved in the shift from endocrine to exocrine secretion of PGF in pigs [80]. In addition, PGE2 and lysophosphatidic acid (LPA), along with its receptor (LPAR3) are important during pregnancy. Expression of PGE2 synthase by trophoblast and endometrium decreases production of PGF to favor PGE2 that supports CL maintenance. In addition, there are increases in LPA in the uterine lumen and LPAR3 on pig conceptuses in response to E2 [81] during early pregnancy. LPA likely induces migration and spacing of pig blastocysts which are critical events preceding implantation and placentation in pregnant pigs [81].

Maternal recognition of pregnancy occurs on Days 11 to 12 in the pig. In cyclic gilts, luteal regression begins on about Day 15 as concentrations of P4 in plasma decline to basal levels by Days 17 to 18. At the same time, OXT is released in a pulsatile manner by the uterus and binds OXTR to stimulate pulsatile release of PGF from uterine epithelia [5]. Regression of CL occurs in response to pulsatile release of PGF into the uterine venous drainage beginning on Days 15 and 16 of the estrous cycle [79]. The amounts of PGF and PGE2 in the uterine lumen are greater in pregnant than cyclic pigs, but uterine PGF is converted into an inactive metabolite through a utero-ovarian countercurrent vascular pathway within the broad ligament, and PGE2 synthase:PGF synthase ratios are higher in CL from pregnant than cyclic pigs, but not between CL ipsilateral or contralateral to the pregnant uterine horn [82].

### Conceptus interferons (IFN) in pigs

Pig conceptus trophectoderm secretes substantial amounts of Type 2 IFNG and lesser amounts of a novel Type 1 IFND between Days 14 to 18 of pregnancy [83]. But, there is little understanding of either autocrine or paracrine effects of IFNG and IFND *in utero*, and they are not detectable in uterine venous blood [83,84]. Intrauterine infusion of total porcine conceptus secretory proteins between Days 12 to 15 of the estrous cycle does not affect the interestrous interval or temporal changes in concentrations of P4 in plasma, but does increase uterine secretion of PGE2 [83,85]. However, these porcine trophoblast IFNs may affect gene expression by uterine epithelia to affect events during the establishment of pregnancy and implantation [17,86].

Blastocyst attachment to uterine LE, a critical step in implantation, is achieved by labilization and remodeling of uterine LE to change its polarity in response to signaling from the conceptus [87]. Pig trophoblast IFNs may affect remodeling and/or depolarization of the uterine epithelia as a prerequisite for implantation and establishment of a functional placenta [17]. On Day 15 of pregnancy, immunoreactive IFNG is present in unevenly distributed clusters in uterine LE [17], as well as cells, likely macrophages, in uterine stroma and STAT1 expression is limited to uterine LE adjacent to pig conceptus trophectoderm expressing *IFNG* mRNA [54]. On Day 15 of pregnancy, zona occludens one (ZO1), a marker of epithelial tight junctions, is detectable on the basal side of uterine LE, suggesting changes in polarity of uterine LE in response to pig conceptus interferons [17]. IFNG is a specific and potent inducer of major histocompatability complex (MHC) class II antigens [84]. Uterine stromal cells of Day 15 cyclic gilts do not express MHC class II antigens (swine leukocyte antigens, SLA), but SLA antigens are abundant in endometrial stroma and within vascular elements beneath uterine LE in pregnant pigs. The uterine LE is completely negative for SLA antigens in pregnant pigs, suggesting that those cells are not receptive to IFNG via classical cell signaling pathways. Alternatively, the presence of IRF2, a potent inhibitor of gene transcription in sheep uteri [88] is induced in uterine LE of pigs by E2 [45] and this likely suppresses expression of SLA antigens in uterine LE of pigs. Accordingly, expression of classical ISGs, including Mx, ISG15/17, IRF1, STAT1 and STAT2 is limited to uterine stromal cells in pigs between Days 14 and 18 of pregnancy [54,86]. Induction of or increased expression of classical ISGs is restricted to stromal cells and GE of sheep, cow, pig, mouse, rat, primates, human, and pig [7]. Emerging evidence suggests that induction of ISGs in the uterus by conceptus IFNs is conserved in early pregnancy in many mammals and this likely facilitates gene expression in uterine epithelia and stromal cells required for implantation and production of histotroph critical to conceptus development. However, definitive effects of IFNG and IFND from porcine trophectoderm on uterine functions are not known.

### Fibroblast growth factor 7, an estromedin in pigs

Pig conceptuses secrete E2 between Days 10 and 15 for pregnancy recognition, but also to increase expression of genes within the uterine LE, which act on conceptus trophectoderm and uterine LE to stimulate proliferation, migration, adhesion and gene expression that supports implantation and development of the conceptus [53]. The limited number of E2-stimulated genes localized in uterine LE of pigs include: *AKR1B1, B2M, CD24, EDG7, FGF7, IRF2, MX1, NMB, SLAs 1, 2, 3, 6, 7, 8, SLC5A1, SPP1,*and *STC1*. Insulin-like growth factor 1 (IGF1) is expressed by uterine glands of cyclic and pregnant pigs and IGF1 receptors are expressed by cells of the endometrium and conceptuses, suggesting paracrine and autocrine actions of IGFI. FGF7 is an established stromal cell derived paracrine mediator of hormone-regulated epithelial growth and differentiation. As noted previously, E2 induces IRF2 in uterine LE/sGE to ensure expression of novel genes by those cells that are in intimate contact with trophectoderm and to restrict expression of classical interferon stimulated genes to uterine GE and stromal cells.

There is novel expression of FGF7 by uterine LE between Days 12 and 15 of the estrous cycle and pregnancy and FGFR2IIIb is expressed by uterine epithelia and conceptus trophectoderm. E2 secreted by pig conceptuses increases *FGF7* gene expression in pigs, but only after P4 has suppressed expression of PGR by uterine epithelia. In turn, FGF7 increases cell proliferation, the abundance of phosphorylated FGFR2IIIb, the MAPK cascade and the expression of plasminogen activator urokinase (PLAU), a marker for trophectoderm cell differentiation. From about Day 20 of pregnancy, FGF7 is expressed by uterine GE in pigs in response to P4 and is presumed to continue to affect uterine epithelia and conceptus development (G.A. Johnson, R.C.Burghardt and F.W. Bazer, unpublished results). Gene expression by cells of the pig uterus during pregnancy and in response to exogenous E2 and/or intra-uterine injections of pig conceptus secretory proteins containing IFNG and IFND have been reported [45,54,78,89-91]. The increased secretion of E2 between Days 15 and 30 of pregnancy also increases expression of endometrial receptors for PRL which is associated with increases in uterine secretory activity [57-60] and uterine blood flow [92].

### Secreted phosphoprotein 1 and pregnancy

#### Sheep

SPP1 is an acidic phosphorylated glycoprotein component of the ECM in epithelia and secretions of many tissues, including the oviduct, uterus, trophoblast and placenta [32]. SPP1 binds to integrin heterodimers including ITGAV:ITGB3, ITGAV:ITGB1, ITGAV:ITGB5, and ITGA4:ITGB1 heterodimers via its arginine-glycine-aspartic acid (RGD) sequence to promote cell adhesion, spreading and migration, as well as calcium transport and phosphotidylinositol 3'-kinase (PIK3) activity [34]. SPP1 increases in abundance in uterine flushings from pregnant ewes between Days 11 to 17 when adherence and attachment of conceptuses to uterine LE occurs [34]. SPP1 then binds integrin heterodimers expressed by trophectoderm and uterus to: 1) stimulate changes in morphology of conceptus trophectoderm; and 2) induce adhesion between uterine LE and trophectoderm essential for implantation and placentation. Although *SPP1* mRNA increases only in GE of pregnant ewes, SPP1 protein is localized on the apical aspect of uterine LE, GE and conceptus trophectoderm. Progesterone induces expression of SPP1 in uterine GE that lack PGR; therefore, the effects of P4 are assumed to be mediated by a P4-induced stromal cell-derived growth factor(s) such as FGF10 and/or HGF in ewes. Administration of both E2 and P4 induces PGR expression in endometrial GE that is followed by a dramatic decrease in expression of SPP1 [49].

#### Pigs

In pigs, *SPP1* mRNA is initially detected in discrete regions of uterine LE juxtaposed to the conceptus just prior to implantation on Day 13. Expression of SPP1 is induced by E2 from conceptuses beginning on Days 11 and 12 to signal pregnancy recognition and expression expands to the entire uterine LE by Day 20 when firm adhesion of conceptus trophectoderm to uterine LE is established [34,93]. *SPP1* mRNA is not present in pig conceptuses [34]. In contrast, SPP1 protein is abundant along the apical surface of uterine LE and trophectoderm cells along the entire maternal:placental interface during pregnancy [34,93]. At this interface there is also expression of multiple integrin subunits that potentially form heterodimeric receptors for SPP1 including ITGAV:ITGB3, ITGAV:ITGB1, ITGAV:ITGB5, and ITGA4:ITGB1 [21,35]. The interaction between the integrin heterodimers and SPP1 likely induces changes in morphology of trophectoderm and mediates adhesion between trophectoderm and uterine LE essential for implantation and placentation [34,93].

Using a porcine trophectoderm cell line (pTr2) and primary porcine uterine epithelial (pUE) cells, it was determined that pTr2 and pUE integrins bind SPP1 directly [21]. The integrins involved were identified as ITGAV and ITGB6 on pTr2 cells and ITGAV and ITGB3 on pUE cells. Using these cell lines, it was also determined that the RGD sequence in SPP1 is required for dose- and cation-dependent attachment, as well as migration of pTr2 cells and pUE cells [21]. SPP1-induced migration of pTr2 cells was blocked with blebbistatin, an inhibitor of myosin II-mediated motor activity. Further, using SPP1-coated microspheres and pTr2 cells, it was determined that co-localization of ITGAV integrin subunit and talin were associated with assembly of focal adhesions at the apical domain of pTr2 cells. These results indicate that SPP1 stimulates migration and attachment of pig trophectoderm by stimulating force-driven, integrin-mediated, focal adhesion assembly and haptotactic migration required for conceptus elongation and implantation [93]. Interestingly, ITGAV:ITGB6 on conceptus trophectoderm binds discretely to only three ECM proteins, each of which is expressed prominently at the conceptus-endometrial interface of pigs, namely SPP1, fibronectin and the latency associated peptide of TGFB [34].

Using porcine chorioallantoic membranes placed in Ussing chambers, we recently reported that SPP1 enhanced placental transport of ions [94]. Specifically, addition of placenta conditioned medium that contained SPP1 increased the transepithelial voltage within 2 min, indicating that SPP1 exerted its effect through a rapid cell signaling mechanism. A possible mechanism may involve phosphorylation of the Na^+^-K^+^-ATP pump and Na^+^-dependent protein transporters. Removal of SPP1 from placenta conditioned medium by immunoprecipitation prevented stimulatory effects of placenta conditioned medium on ion transport across the placenta. Addition of recombinant rat SPP1 to Ussing chambers also increased ion transport similar to pig placenta conditioned medium. However, recombinant rat SPP1 with a mutated (arginine:glycine:aspartic acid) RGD sequence, which cannot bind integrins, had no effect on ion transport. Thus, we discovered a novel role for SPP1 at the uterine-placental interface of pregnancy. SPP1 is synthesized and secreted from uterine epithelia, binds to integrins on the chorionic epithelium and activates ion transporters to increase nutrient transport across the chorionic membrane to the placental vasculature. Therefore, the strategic localization of SPP1 at sites of active nutrient transport in a variety of placentae may play a crucial role in successful pregnancy outcome in mammals.

### Servomechanism involving E2, P4, IFNT, placental lactogen (CSH1) and placental growth hormone (GH1) in the ovine uterus

During estrus, diestrus and pregnancy, ovine and bovine uteri are exposed sequentially to E2, P4, IFNT, CSH1 and GH1 which initiate and maintain endometrial gland morphogenesis and differentiated cell secretory functions [8]. Placentae of many species, including rodents, humans, nonhuman primates, and ruminants, secrete hormones structurally related to pituitary PRL and GH1 that are termed CSH1 (placental lactogen) [42]. Ovine CSH1 is produced by trophoblast giant BNC from Days 15 to 16 of pregnancy which is coordinate with onset of expression of serine protease inhibitor, kunitz-type 1 (*SPINT1*), *SPP1GRP* (gastrin-releasing peptide), and *STC1* which are excellent markers for GE differentiation and secretory function during pregnancy in sheep [42]. A homodimer of the PRLR (prolactin receptor), as well as a heterodimer of PRLR and GHR (growth hormone receptor), transduce signals by ovine CSH1 [47]. In the ovine uterus, *PRLR* gene expression is unique to GE. Temporal changes in circulating levels of CSH1 are correlated with endometrial gland hyperplasia and hypertrophy and increased production of SPP1 and SPINT1 during pregnancy. Sequential exposure of the pregnant ovine endometrium to P4, IFNT, and CSH1 appears to activate and maintain endometrial remodeling, secretory functions of GE, and perhaps uterine growth during gestation. Chronic treatment of ovariectomized ewes with P4 induces *SPP1UTMP* and *STC1* expression by uterine GE [8]. However, intrauterine infusions of CSH1 further increases *SPP1STC1*, and *UTMP* gene expression in the ovine uterus, but only when ewes receive P4 daily and intrauterine infusions of IFNT between Days 11 and 21 after onset of estrus [49]. The effects of IFNT may be attributed, in part, to increasing *PRLR* in the endometrial glands. Thus, placental hormones play key roles in stimulating endometrial gland morphogenesis and differentiated functions during pregnancy that are required for conceptus development in ruminants. Similar servomechanisms are proposed for the human trophoblast and endometrium during early pregnancy [95].

Endometrial expression of ESR1, PGR and OXTR is not affected by CSH1 or GH1 from the ovine placenta. However, expression of SPINT1 by uterine GE and the density of GE in the stratum spongiosum of the uterine endometrium are increased by both CSH1 and GH1. This effect requires that ewes receive daily intramuscular injection of P4, as well as intra-uterine injections of IFNT between Days 11 and 21, and then intra-uterine injections of CSH1, GH1, or CSH1 and GH1 from Days 16 to 29 after onset of estrus [49]. Only CSH1 stimulates expression of both SPINT1 and SPP1 in uterine GE. As for the peri-implantation period of pregnancy, down-regulation of epithelial PGR is a prerequisite for induction of expression of *SPP1SPINT1* and *STC1* genes in uterine GE by sequential intra-uterine injections of IFNT and either CSH1 or GH1. However, when treated with both P4 and E2, E2 increases PGR in uterine GE which inhibits expression of both SPP1 and SPINT1 proteins. These results indicate that effects of P4 are mediated via one or more progestamedins, e.g., FGF10 and/or HGF from PGR-positive uterine stromal cells in ewes. Further, abrogation of expression of *SPINT1* and *SPP1* genes by uterine GE ceases when ESR1 and PGR are expressed in uterine GE during the peri-parturient period [49].

The concept of a servomechanism in pigs is based on evidence that E2 induces PRLR in uterine LE and GE for shifting secretion of PGF from an endocrine to an exocrine direction for pregnancy recognition [80]. As well, there are synergies between P4, E2 and PRL from the maternal anterior pituitary gland that allow for greater secretory activity of uterine GE in pigs [57-60].

### Growth and development of the conceptus during gestation

#### The Ewe

**Figure 4:**
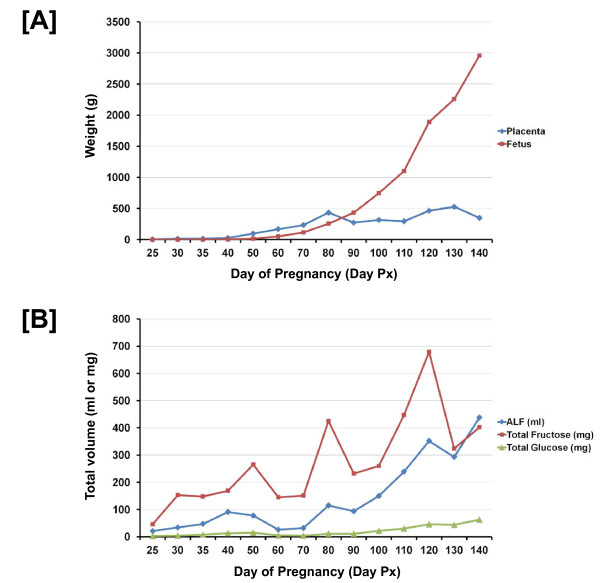
**[A] During pregnancy in ewes the increases in placental length and weight precede the period of rapid growth of the fetus during gestation because the placenta must be established to transport nutrients and gases to the conceptus to ensure it growth and survival.[B]** The comparison of total amounts of glucose and fructose in allantoic fluid (ALF) fluid suggest that fructose is a major substrate for metabolism via the pentose phosphate pathway, hexosamine pathway and glycolytic pathways.

Placental growth and development precedes that of fetal growth in ewes (Figure 4). This is because placental transport of nutrients, oxygen and other trophic factors is essential for fetal growth and development [96]. Placental length is influenced by the extent to which the trophectoderm elongates between Days 12 and 25 of gestation. There are also increases in both vasodilation of blood vessels and growth of new blood vessels (angiogenesis) in placentomes of ewes to support rapid fetal growth as pregnancy advances. There is also development of functional areolae that transport secretions from uterine glands across the placenta for release into the fetal circulation. The composition of uterine gland secretions has been characterized only partially, but they include SPINT1, LGALS15, STC1, GRP, and SPP1 proteins that are secreted by uterine GE in response to P4, GH1 and CSH1. Secretions of ovine uterine glands contain a number of other enzymes, regulatory molecules, growth factors, cytokines, lymphokines and nutrients critical to growth and development of the fetus.

The ovine placenta has, on average, around 70 functional caruncles that interdigitate with corresponding placental cotyledons to form placentomes for the exchange of micronutrients, e.g., amino acids and glucose, as well as gases, between the vascular systems of the conceptus and ewe. The number of functional placentomes is variable among ewes; however, if a ewe has a marginal number of placentomes, there is often compensatory growth of the placentomes that are present so that fetal weight is not necessarily affected. The basis for failure of some caruncles to develop into functional caruncles and, in turn, placentomes may be due to the so-called “field effect.” The “field effect” is that gradients exist in the degree of differentiation of tissues such as the mammary gland. This is most evident in pigs as the dominant piglets nurse the middle and anterior teats that produce more milk than those located near the inguinal area.

There is the perception that the allantoic sac is a reservoir for fetal waste; however, the allantois is, in fact, a reservoir for nutrients. Indeed, rapid transport of water into the allantois expands it so that it fuses with the chorion to form the chorioallantoic placenta [97]. The volume of allantoic fluid increases in ewes from Day 25 (21 mL) to the first peak on Day 40 (91 mL), decreases to Day 70 (32 mL) and then increases to Day 140 (438 mL) of the 147 day period of gestation. This pattern of change in allantoic fluid volume is similar to that for pigs between Days 20 and 30 of gestation, but a second major peak in allantoic fluid volume between Days 55 and 70 in pigs is followed by a steady decline to term (Day 114 in pigs) [97].

### Nutrients in Fetal Fluids

Concentrations of glucose and total amounts of glucose in allantoic fluid are affected very little due to day of gestation; however, concentrations of fructose and total fructose in allantoic fluid are much greater and change significantly with day of gestation in ewes. The role(s) of fructose in conceptuses of livestock species and other mammals with epitheliochorial and syndesmochorial placentae that are fructogenic is not known.

Fructose is the most abundant hexose sugar in fetal fluids of ungulate mammals [98,99]. In general, high levels of fructose are found in fetal blood and fetal fluids of mammals having epitheliochorial and synepitheliochorial placentae which contain little or no glycogen [98]. Studies of pregnant ewes revealed that: 1) intravenous administration of glucose into ewes results in a rapid increase in glucose followed by a protracted increase in fructose in fetal blood; 2) injection of glucose into the umbilical vein of the fetus increases glucose in maternal blood and hyperfructosemia in the fetus indicating that glucose can move from conceptus vasculature to maternal blood, whereas fructose derived from glucose is not transported into maternal blood; 3) the placenta is the site of conversion of glucose to fructose; 4) fructose is continuously produced by the placenta independent of glucose concentration in maternal or fetal blood; and 5) the flux of glucose from the maternal to the fetal circulation can be as much as 70 mg/min in ewes made hyperglycemic [100,101]. These results were confirmed in studies using radiolabeled glucose to demonstrate its conversion to radiolabeled fructose by the placenta of pigs [102,103].

The role of fructose is not known since it has not been the subject of studies to determine its role in metabolic pathways except for those indicating that it is not metabolized via the glycolytic pathway or Krebs cycle [101,104-106]. Nevertheless, fructose can be utilized for synthesis of nucleic acids and generation of reducing equivalents in the form of NADPH H^+^ in the fetal pig [103] and in HeLa cells [105]. However, there are reports that neither fructose nor glucose is metabolized via the pentose phosphate pathway in the ovine placenta [104,107]. Fructose and glucose are equivalent in entering metabolic pathways leading to synthesis of neutral lipids and phospholipids in heart, liver, kidney, brain and adipose tissue of fetal lambs which refutes general statements that fructose in not metabolized in fetal tissues of domestic animals [108]. The activities of glucose-6-phosphate dehydrogenase, malic enzyme and acetyl-CoA carboxylase in liver are stimulated by glucose in adult rats which increases lipogenesis [109] and fructose enters adipocytes by both insulin-independent and insulin-insensitive mechanisms[110].

It is of interest that researchers focused on intra-uterine growth restriction (IUGR) as well as subsequent adult onset of metabolic disease in various ungulate species have not considered fructose to be an important metabolic substrate. This seems to be so because fructose is not metabolized via the glycolytic pathway or Kreb’s cycle in the placenta, fetus or neonate [101,104-106,111,112]. In ewes, for example, the maximum concentration of glucose in allantoic fluid is 1.1 mmol/L between Days 35 and 140 of pregnancy, whereas the concentration of fructose is between 11.1 and 33 mmol/L during the same period of pregnancy [96]. Therefore, fructose is exerting effects on cell proliferation at molar concentrations well below those in allantoic fluid. Glucose, on the other hand, exerts effects at concentrations well above those in allantoic fluid [96]. Fructose may be the most likely hexose sugar to stimulate MTOR nutrient sensing cell signaling and synthesis of glycosaminoglycans from fructose and glutamine (another abundant nutrient in porcine and ovine allantoic fluids) via the hexosamine pathway to stimulate growth and development of the conceptus.

Fructose is also the primary sugar in blood, allantoic fluid and amniotic fluid of the fetal pig to about Day 80 of gestation, but it decreases thereafter as glucose increases between Days 82 and 112 of the 114 day period of gestation [113]. The rapid clearance of fructose from blood of piglets by 24 h post-partum indicates that the neonatal piglet is unable to utilize fructose as an energy source [99,114].

**Figure 5:**
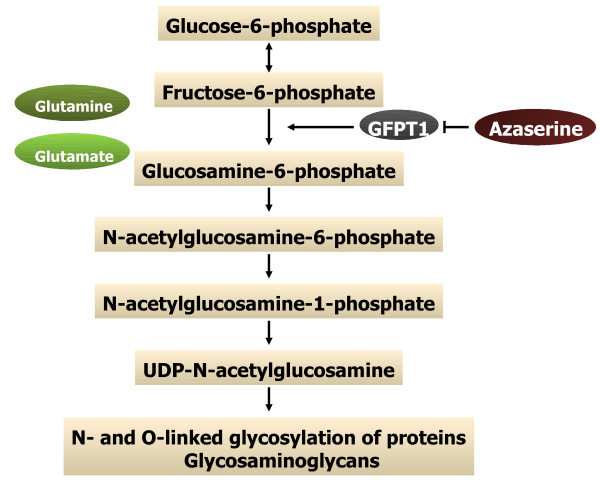
The hexosamine pathway allows for both glucose and fructose to be metabolized to glucosamine-6-phosphate that lead to activation of the MTOR cell signaling pathway, as well as synthesis of glycosaminoglycans such as hyaluronic acid, that are critical to growth and development of the conceptus.

Based on the lack of understanding of the role of fructose, the most abundant hexose sugar in the pregnant uterus, we conducted experiments to discover that fructose is actively involved in stimulating cell proliferation and mRNA translation via activation of MTOR cell signaling and synthesis of glycosaminoglycans via the hexosamine metabolic pathway [115]. Glucose induces proliferation of human trophoblast cells through MTOR signaling in a PI3K-independent mechanism that involves activation of MTOR by metabolites of the GFPT1 pathway, particularly UDP-N-acetylglucosamine (GlcNAc) [116]. UDP-GlcNAC is responsible for phosphorylation of TSC2, a GTPase activating protein, and p70S6K1, a protein kinase downstream of MTOR, to stimulate trophoblast cell proliferation in response to metabolism of glucose to glucose-6-PO_4_, fructose-6-PO_4_ and glucosamine-6-PO_4_. Glucose and fructose can also be used in the hexosamine pathway for synthesis of hyaluronic acid that can affect angiogenesis and other aspects of fetal-placental development during pregnancy (Figure 5). The pig placenta contains significant amounts of hyaluronic acid and hyaluronidase [117], both of which increase in the uterine lumen of pigs in response to progesterone [118]. Hyaluronic acid may stimulate angiogenesis and/or stimulate angiogenesis, morphogenesis and tissue remodeling of the placenta as reported for the human placenta [119]. The accumulation of Wharton’s Jelly occurs in the placentae of most mammals and localizes to the umbilical cord primarily, but to a lesser extent to placental blood vessels [120] and it is composed primarily of hyaluronic acid that also supports fibroblasts and stem cells [121]. It is clear that angiogenesis is critical to conceptus development in all species and results of the present study indicate that fructose is used for synthesis of glycosaminoglycans such as hyaluronic acid that support angiogenesis, particularly in the placenta.

There is altered glucose metabolism in ewes with fetuses that experience intrauterine growth retardation due to placental insufficiency which affects concentrations of myo-inositol, sorbitol and fructose. The redirection of placental glucose into myo-inositol is likely due to decreased sorbitol and fructose production within the placenta via aldose reductase that requires NADPH.

The abundance of fructose is likely due to high hepatic sorbitol dehydrogenase activity and high placental aldose reductase activity for conversion of glucose to sorbitol. Glucose is transported into and out of cells by both facilitative (e.g., SLC2A) and sodium-dependent (e.g., SLC5A) transporters. The glucose transporters SLC2A1 and SLC5A1 are most abundant in ovine endometria and SLC2A1, SLC2A3, SLC2A4, SLC5A1 and SLC5A11 are most abundant in trophectoderm and endoderm of ovine conceptuses. A portion of glucose transported into trophoblast cells is converted to fructose which is unable to return to the maternal circulation, but does enter the fetal circulation. Fructose may be converted to fructose-6 phosphate and then to glucosamine-6-phosphate by glutamine:fructose-6-phosphate amidotransferase 1 (GFPT1). Glucosamine-6-phosphate is required for production of glycosaminoglycans such as hyaluronans necessary for formation of the fetal-placental stroma and fetal-placental development. Although fructose is not an energy source for fetal-placental tissues of pigs or sheep, it may regulate the mechanistic target of rapamycin (MTOR) cell signaling pathway to regulate proliferation, migration and mRNA translation in trophectoderm cells of sheep and pigs.

Changes in concentrations of electrolytes, particularly sodium:potassium ratios and allantoic fluid volume increase coordinately between Days 25 and 40 of gestation in pregnant pigs, whereas a decrease in the sodium:potassium ratio is associated with increases in allantoic fluid volume between Days 60 and 147 of gestation in ewes. In pigs the sodium:potassium ratio in allantoic fluid is greater than 1 when allantoic fluid volume increases between Days 20 and 30 and again between Days 40 and 60 of gestation, but are less than one when allantoic fluid volumes decrease between Days 30 and 40 and after Day 60 of gestation [122]. There are higher concentrations of sodium and lower concentrations of potassium with sodium:potassium ratios of about 10 in cows with hydroallantois compared to cows with normal volumes of allantoic fluid and sodium:potassium ratios of 1 to 2. Further, increases in allantoic fluid volume between Days 60 and 140 of gestation is coordinate with increasing concentrations of placental lactogen in maternal blood of ewes. Both human placental lactogen and prolactin stimulate short circuit current and potential difference across human and pig placentae that increase sodium-dependent transport of water and other actively transported molecules.

Volume and composition of amniotic fluids change during the course of gestation in ewes. Amniotic fluid serves several important roles: 1) it buoys the fetus to allow it to develop symmetrically in three dimensions; 2) it prevents fetal skin from adhering to the amnion; and 3) it is a source of nutrition as the fetus drinks up to 1 liter of amniotic fluid per day during the last one-third of gestation. As for allantoic fluid, changes in volume and content of glucose in amniotic fluid are associated with changes in electrolytes and their ratios. Temporal changes in concentrations of progesterone, estradiol, estrone and estrone sulphate in maternal blood, fetal blood and allantoic fluid are similar during the course of gestation in ewes. The placenta produces significant amounts of sex steroids in pregnant ewes.

Concentrations of amino acids in maternal plasma of sheep do not vary substantially during pregnancy [123]. However, there are marked changes in concentrations and total amounts of amino acids in ovine allantoic and aminotic fluids between Days 30 and 140 of gestation [123]. Specifically, concentrations of alanine, citrulline, and glutamine in allantoic fluid increase 20-, 34-, and 18-fold, respectively, between Days 30 and 60 of gestation, and are 24.7, 9.7, and 23.5 mmol/L, respectively, on Day 60 of gestation. Remarkably, alanine, citrulline (the immediate precursor of arginine) plus glutamine (a precursor for citrulline synthesis) account for about 80% of total α-amino acids in allantoic fluid during early gestation. Notably, serine (16.5 mmol/L) contributes approximately 60% of total α-amino acids in allantoic fluid on Day 140 of gestation. These findings of the unusual abundance of traditionally classified nonessential amino acids in allantoic fluid raise important questions regarding their roles in ovine conceptus development.

### Conceptus development in the pig

The desired effect of placentation in pigs is to achieve contact with a maximum amount of uterine endometrial surface area for the exchange of nutrients and gases across a true epitheliochorial placenta in which maternal blood and fetal blood is separated by 6 layers of cells. Under conditions of intra-uterine crowding, there is a different pattern of embryonic death loss in swine [97]. For intact gilts and sows, the major portion of embryonic mortality in swine occurs prior to Day 25 of gestation, but for gilts subjected to superovulation, embryo superinduction and unilateral ovariectomy-hysterectomy, intra-uterine crowding results in a high percentage of fetal deaths with a particularly high incidence of dead and reabsorbing fetuses between Days 40 and 70 of gestation. The two major factors regulating prenatal survival in swine are: 1) endometrial surface area available for placentation; and 2) the extent of development of the blastocysts during the peri-implantation period of pregnancy when the extent of development of the placental membranes is established.

Placental surface area is influenced greatly by the rapid increase in allantoic fluid volume between Days 20 and 30 of pregnancy which is followed by a decrease in allantoic fluid volume to Day 40, a second increase to Day 60 and a then a decrease to Day 100 of pregnancy [97]. The rapid increase in allantoic fluid volume between Days 20 and 30 of gestation expands the chorioallantoic membranes and allows them to establish intimate contact with a maximum amount of endometrial surface. At Day 30 of gestation, allantoic fluid volume is highly positively correlated with placental length, placental weight and allantoic fluid estrone concentration. Also, during the second period of accumulation of allantoic fluid between Days 40 and 60 of pregnancy, allantoic fluid volume is highly correlated with placental length and weight and total protein in allantoic fluid. Osmotic gradients across the chorioallantois result in the rapid accumulation of water in the allantoic sac, along with glucose, fructose, amino acids, polyamines and many proteins secreted by the uterine glandular epithelia and transported into the allantoic fluid by placental areolae that number about 2,500 per placenta in pigs. Placental areolae appear initially in greatest concentration in the interior sections of the placentae and then developed toward the polar sections so that by Day 50 of gestation there was no significant difference in the number of areolae between the two areas of the placentae. Areolae surface area in both interior and polar sections of placentae, total number of areolae per placenta and total areolae surface area per placenta are greater for intact versus UHOX gilts. The total numbers of areolae increase from Day 25 to 30 to Day 50 of pregnancy and then remain relatively constant thereafter. Total areolae surface area also increases rapidly to Day 50 of gestation and then increases slowly, but continuously, to Day 100 of pregnancy.

Average placental length increases rapidly between Day 20 (7.3 cm) and 30 (49.6 cm) of gestation and continues to increase to Day 60 of pregnancy, but changes little thereafter. Placental weight also increases from Day 30 (27.6 g) to Day 100 (258.0 g). The increase in placental length precedes the increase in placental weight and placental weight and length change little after Day 60 of gestation. Placental surface area also increases between Days 30 and 70 of pregnancy, but changes little thereafter. However, capillary bed volume in the pig placenta continues to increase until term due to on-going angiogenesis in the allantoic membrane. The most rapid increase in fetal weight occurs after Day 50 of pregnancy when placental development is essentially completed. Intra-uterine crowding and the associated decrease in endometrial surface area inhibits placental development and, in turn, increases fetal mortality and retards development of those fetuses which survive. Placental weight is as good a predictor of fetal wet weight and fetal survival as any combination of placental variables. It is clear that placental weight and fetal weight are highly correlated in the latter stages of pregnancy in swine [97].

Amniotic fluid volume also changes during gestation, but measurable amounts (e.g., 0.2 mL in a live embryo on Day 25) are present prior to Day 30 of gestation. Amniotic fluid volume then increases from Day 30 to Day 70, plateaus to Day 80 and then decreases to Day 100. Maximum amniotic fluid protein concentration occurs on Day 60 of gestation and maximum amniotic fluid total protein is present on Day 70 of gestation [97].

Concentrations of sex steroids change dramatically in maternal and fetal blood and in allantoic fluid during the course of gestation in pigs [97]. As early as Day 14 of pregnancy, pig conceptuses convert progesterone, androstenedione, and dehydroepiandrosterone to estrone and estradiol. The increase in concentrations of estrogen between Days 20 and 30 of gestation is associated with water imbibition by uterine and placental tissue and increased uterine blood flow, both of which are critical to provide adequate oxygen for the rapidly developing placenta and fetus. The rapid increase in estrogen secretion by placentae between Days 60 and 100 of gestation is coordinated with increases in transport of amino acids and sugars into the pregnant uterus.

Results presented here on aspects of conceptus development in ewes and pigs provide a benchmark for studies examining effects of nutrition, environment, genotype, epigenetics, and other factors in ewes. Currently, studies are underway to advance understanding of mechanisms responsible for changes in water and electrolytes, transport of sugars, proteins and sex steroids, and formation and growth of the placenta. These physiological processes underpin growth, development and survival of the conceptus and ensure successful outcomes of pregnancy.

### Nutrients for enhancing growth and survival of conceptuses

An important advance in improving the survival and growth of mammalian embryos and fetuses resulted from our discovery of an unusually high abundance of arginine, ornithine and glutamine in porcine allantoic fluid during early gestation [124]. Specifically, on Days 40 of gestation (term = 114 days), concentrations of arginine in porcine allantoic fluid are 4 to 6 mmol/L, when compared with its maternal plasma levels (0.1 to 0.14 mM). In addition, there are particularly high concentrations of ornithine (1 to 3 mmol/L; a metabolite of arginine) and glutamine (3 to 4 mmol/L; a precursor of arginine) in porcine allantoic fluid on Day 40 of gestation, when compared with maternal plasma levels (0.05 to 0.1 mmol/L for ornithine and 0.3 to 0.45 mmol/L for glutamine). Remarkably, concentrations of arginine, ornithine, and glutamine in porcine allantoic fluid increase by 23-, 18-, and 4-fold, respectively, between Days 30 and 40 of gestation, with their nitrogen accounting for 67% of the total free α-amino acid nitrogen. The unusual abundance of the arginine-family of amino acids in fetal fluids is associated with maximal placental NO and polyamine syntheses in the first half of pregnancy when placental growth is most rapid. Physiological levels of both NO and polyamines are crucial to placental angiogenesis and growth, as well as embryonic and fetal growth and survival. These novel findings suggest that arginine-dependent metabolic pathways play an important role in growth and development of the porcine conceptus [125-127]. In support of this notion, dietary supplementation with 1.0% arginine-HCl between Days 30 and 114 of gestation increased: (a) concentrations of arginine, ornithine, and proline in plasma by 77%, 53%, and 30%, respectively, and (b) the number of live-born piglets by 2 and litter birth-weight by 24% [128].

### Concepts of tensegrity applied to mechanosensation and mechanotransduction within the uterine wall during the post-implantation period of pregnancy

Mechanical forces directly affect the form and function of cells, tissues and organs [129]. No other organ adapts to greater changes in mechanical forces than the uterus that is subjected to pregnancy-induced stretch [130]. The uterine wall consists of mechanically dissimilar tissue compartments including epithelial, endothelial, stromal, smooth muscle (myometrial) and immune cells, which are each surrounded by distinctive ECM and respond differently to stretch. The mechanical properties of cells and their surrounding ECM are essential to the mechanisms by which cells sense forces (mechanosensation), transmit them to the cell interior through integrins and/or other adhesion molecules, and transduce them into diverse biochemical signals (mechanotransduction). Some of these signals impact cytoskeletal responses that generate intracellular tension necessary to balance external forces. The biochemical signals generated can then impact the function and effectiveness of other stimuli such as hormones, growth factors and nutrition [131]. The uteri of pregnant sheep have provided a unique model to develop important insights into force-induced tissue remodeling.

**Figure 6:**
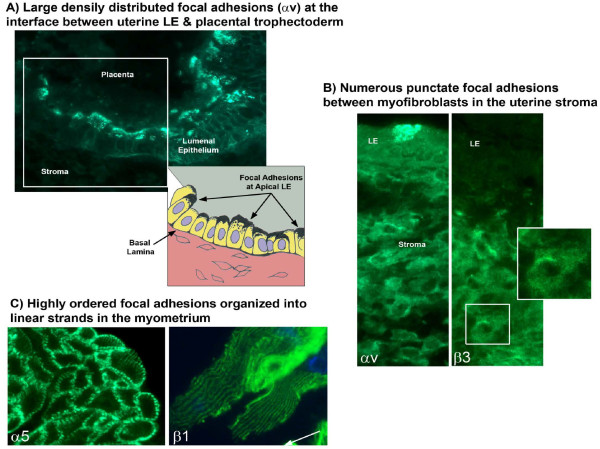
**Focal adhesions are dramatically upregulated in different tissue-level compartments within the uterus during pregnancy in response to continuous application of local force outside the cell balanced by cytoskeletal contraction.** The different organization and size of these macromolecular assemblies containing cytoskeletal binding proteins, adapter proteins and signaling molecules illustrate how mechanical forces regulate the form and function of the uterine wall and also reveal markedly different elastic microenvironments.

As noted in a previous section, it is well established that multiple integrin components of histotroph, including ECM proteins, play an important role in conceptus attachment and elongation; the focal adhesions that assemble represent a point of convergence for multiple signaling pathways. However, as pregnancy progresses, alterations in the structure and size of focal adhesions reflect tissue compartment-specific adaptation to increasing force and tension exerted by the growing fetus and increasing fetal fluids. Recent studies in sheep reveal that as pregnancy progresses, ECM (mostly SPP1) at the interplacentomal maternal-conceptus interface exhibits increased rigidity and/or transmits increased force that is balanced by cytoskeletal tension (Figure 6). This is reflected in the assembly of progressively larger and more densely distributed aggregates of integrin subunits αv (ITGAV), α4 (ITGA4), α5 (ITGA5), β1 (IGTB1) and β5 (IGTB5) (but not β3 [IGTB3]) that co-distribute with mechanosensory and signal generating focal adhesion constituents (i.e., α-actinin [ACTN] and Y392-phosphorylated focal adhesion kinase [PTK2], respectively, along interplacentomal uterine LE and trophectoderm cells where they attach to one another [34,132]. Large stress fibers composed of bundles of actin filaments crosslinked together by α-actinin and anchored to apical focal adhesions extend deeply into both LE and trophectoderm cells. There is an anisotropic distribution of focal adhesions at mid pregnancy that becomes evenly distributed along most of the maternal conceptus interface by Day 120 of pregnancy indicative of increasingly uniform stretch and/or stiffness of the uterine wall. In contrast placentomes, which contain a highly convoluted and interdigitated syncytial caruncular-cotlyedonary interface that physically dissipates external force and tension, exhibit dramatically smaller and more diffusely distributed aggregations for each of these integrin subunits.

Following firm attachment of the conceptus, fibroblasts within the subepithelial stroma undergo hypertrophy and hyperplasia and differentiate into myofibroblasts that have a phenotype that is intermediate between fibroblasts and smooth muscle cells. This process is identical to that observed during the active remodeling of ECM that occurs in wound healing [132]. Myofibroblasts accumulate the alpha isoform of smooth muscle actin (αSMA) and organize robust stress fibers only when they encounter stress/forces that result from tension or increased rigidity of the ECM. In invasive species (e.g., primates) the expression of αSMA accompanies the decidual reaction [133,134]. However, myofibroblasts also develop in noninvasive species including sheep which do not decidualize. During sheep pregnancy, myofibroblasts begin to differentiate around Day 25 and these cells also express high levels of transforming growth factor beta (TGFB) (G. Johnson, unpublished results), the αv and β3 integrin subunits that are localized to numerous punctate focal adhesions, and a variety of ECM proteins including fibronectin, vitronectin, and SPP1 [34,135]. SPP1 and the latency associated peptide of TGFβ are ligands for αv integrins, and their interaction with integrins contributes to the amplification of mechanical signals that promote force balance by reorganizing the ECM into a more rigid state aligned with focal adhesion formation and increased contractile functions of the cytoskeleton [136,137]. Cell-generated tension contributes to the release of TGFβ from rigid ECM that maintains the contractile phenotype. Parturition results in the elimination of mechanosensory stimuli leading to dedifferentiation of myofibroblasts and turnover of cytoskeletal and ECM proteins that contribute to force balance.

Smooth muscle cells of most hollow organs exhibit a highly ordered structures originally identified as smooth muscle adherens junctions that represent an unusual type of focal adhesion organized into linear strands containing the α5β1 integrin linking the mechanosensory ECM protein fibronectin (FN) with many of the cytoskeletal proteins and signaling molecules that assemble within cells upon integrin activation [138]. However, studies in rodents have shown that focal adhesions in uterine smooth muscle cells are transiently expressed during pregnancy when both hormones of pregnancy and mechanical stretch upregulates the expression of α5β1, fibronectin, and other mechanosensory and signal generating focal adhesion constituents [139,140]. The development of focal adhesions during pregnancy contributes to development of a strong linkage between cytoskeletal proteins and the ECM needed to generate contractile activity sufficient to expel term fetuses at labor [140]. A similar process of focal adhesion in takes place in sheep myometrium, however, initial assembly of focal adhesions occurs near the end of the first trimester of pregnancy but continues to increase in organization as mechanical stretch of the uterine wall results from growth of the fetus and accumulation of fetal fluid. Recent studies in human myometrium have identified a direct link between stretch activated focal adhesion proteins and the activation of myometrial contractility [130]. Collectively, the analyses of different tissue-level compartments within the uterus of the pregnant ewe illustrate how mechanical forces regulate the form and function of the uterine wall during tissue remodeling and also reveal their markedly different elastic microenvironments.

### Ethical approval

This is a review paper; however, all results reported based on research by the authors was approved by the Texas A&M University Animal Care and Use Committee.

## Competing interests

The authors declare that they have no competing interests.

## Authors’ contributions

FWB, GS, JK, KAD, MCS, GAJ, RCB and GW contributed to the writing of this review paper. All authors read and approved the manuscript.
